# Takotsubo Cardiomyopathy in the Absence of an Identifiable Emotional Stressor

**DOI:** 10.7759/cureus.66923

**Published:** 2024-08-15

**Authors:** Katerina Bello, Carla Rabassa

**Affiliations:** 1 Medical School, Herbert Wertheim College of Medicine, Miami, USA; 2 Internal Medicine, Herbert Wertheim College of Medicine, Miami, USA

**Keywords:** stress-induced cardiomyopathy, clinical case report, chest pain, hypokinesis, takotsubo cardioyopathy

## Abstract

This is a case presentation of a 61-year-old female with a history of long-term asymptomatic left bundle branch block and recurrent nephrolithiasis who presented to the emergency department with chest pain that radiated to the left shoulder and jaw, nausea, vomiting, and generalized weakness. On admission, the electrocardiogram showed prolonged QRS complex, significant T-wave inversions in leads V2-V4, and left bundle branch block. Troponin I serum levels were found to be markedly elevated. The echocardiogram demonstrated left ventricular hypokinesis. The patient was admitted for treatment of non-ST-elevation myocardial infarction and was placed on a heparin drip with daily aspirin and high-intensity statin. Cardiac catheterization showed angiographically normal coronary arteries with no signs of obstruction or stenosis. Upon questioning, the patient did not endorse any recent emotionally or physically triggering incidents. Despite the lack of an identifiable emotional stressor, the patient met the diagnostic criteria for takotsubo cardiomyopathy (TTC) and was subsequently placed on evidence-based medical therapy. While most individuals with TTC will fully recover their cardiac function with proper treatment, a subset of patients may continue to have symptoms of persistent heart failure following their initial diagnosis. The pathophysiology of TTC is still not well understood. While the leading theory describes a catecholamine surge secondary to an emotionally or physically triggering event causing myocardial injury and subsequent temporary cardiac dysfunction, further research must be done to understand the underlying pathophysiology of this condition fully.

## Introduction

Takotsubo cardiomyopathy (TTC), otherwise known as stress cardiomyopathy or “broken heart syndrome”, is a reversible condition characterized by transient left ventricular systolic dysfunction. This condition received its characteristic name from the Japanese word for an octopus trap, a “takotsubo”, which resembles the systolic apical ballooning of the left ventricle typically seen in this unique form of cardiomyopathy [1]. TTC often presents with clinical features that mimic those of myocardial infarction (MI) including chest pain and dyspnea. Moreover, electrocardiogram (EKG) findings in patients with TTC frequently exhibit ischemic changes (e.g., ST elevations, ST depressions), further complicating the initial differentiation between TTC and MI. However, a key feature distinguishing these conditions from one another is the lack of coronary artery obstruction or plaque rupture noted in patients with TTC. Only about 2% of patients who are initially suspected to have acute coronary syndrome are eventually diagnosed with TTC [2], making it quite a rare diagnosis. While the pathophysiology of TTC is still not fully understood, the leading theory describes a catecholamine surge following a stressful or emotionally charged event that leads to myocardial injury and temporary dysfunction [3]. Due to the similar presentation of TTC and MI, patients with TTC are often not diagnosed until after they have undergone a cardiac catheterization with no findings of coronary artery obstruction. The purpose of this report is to present a case of TTC where no emotional or physical trigger was identified, highlighting the need for further research regarding the pathogenesis of this condition. Additionally, we hope to urge clinicians to consider TTC as a differential diagnosis in patients presenting with chest pain or other symptoms consistent with MI, even in the absence of an identifiable emotional or physical stressor.

## Case presentation

A 61-year-old Hispanic female presented to the emergency department (ED) complaining of persistent chest pain with intermittent radiation to the left shoulder and jaw, nausea, vomiting, and generalized weakness. Her symptoms began the day prior after completing light yard work and moving furniture inside her home. Further questioning regarding the patient’s history revealed that she was diagnosed with an asymptomatic left bundle branch block many years ago found incidentally on a preoperative EKG. Per the patient, she was never worked up for this diagnosis and therefore has no knowledge of any underlying cardiac condition. She never experienced any symptoms or complications related to this diagnosis. Family history was significant for MI in her father. Upon arrival at the ED, she was found to be hemodynamically stable and afebrile. Vitals were as follows: Temperature: 36.7°C; heart rate: 85 bpm; respiratory rate: 18; blood pressure: 106/64; oxygen saturation: 98%. Physical examination in the ED was entirely unremarkable.

**Figure 1:**
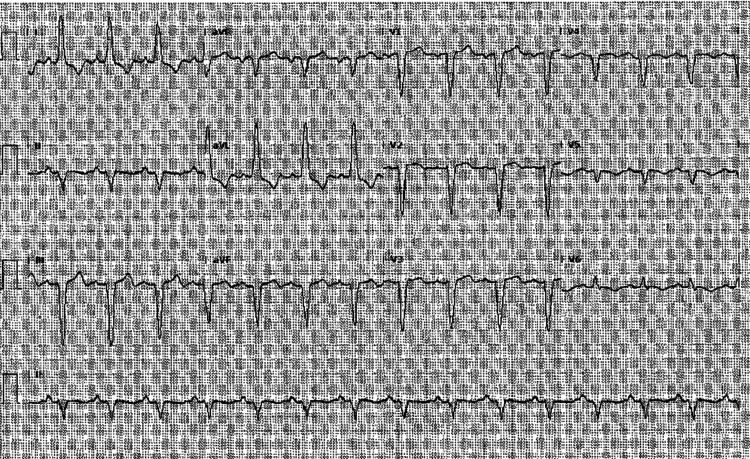
12-lead EKG showing prolonged QRS complex, significant T-wave inversions in leads V2-V4 without reciprocal changes, and left bundle branch block.

An EKG performed in the ED (Figure 1) showed prolonged QRS complex, significant T-wave inversions in leads V2-V4 without reciprocal changes, and left bundle branch block.

The initial high sensitivity troponin level was 9,592 ng/dL (normal range: 0-54 ng/L) with a repeat value of 11,398 ng/L three hours later. Other notable laboratory results included a white blood cell count of 11.46 k/uL (normal range: 3.40-11.00 k/uL) and a potassium level of 3.2 mmol/L (normal range: 3.7-5.1 mmol/L). Coagulation studies and urinalysis were within normal limits. Chest X-ray and the abdominal CT scan without contrast were negative. The patient was admitted for treatment of non-ST-elevation myocardial infarction. She was placed on a heparin drip, aspirin, and a high-intensity statin with cardiac catheterization scheduled.

**Figure 2:**
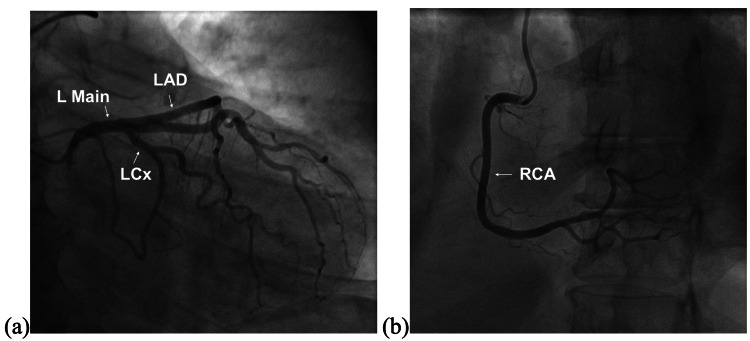
Coronary angiogram depicting (a) left coronary artery and branches and (b) right coronary artery and branches free of disease, obstruction, or stenosis. L Main: left main coronary artery; LAD: left anterior descending artery; LCx: left circumflex artery; RCA: right coronary artery

Within a few hours of starting this regimen, the patient's chest pain had improved. An echocardiogram demonstrated reduced left ventricular systolic function with an ejection fraction of 40-45% with mild to moderate mitral regurgitation and moderate tricuspid regurgitation. Global hypokinesis was noted, including left ventricular apical akinesis, distal septal wall hypokinesis, apical lateral wall hypokinesis, apical inferior wall hypokinesis, and apical anterior wall hypokinesis. Subsequent cardiac catheterization (Figure 2) found the patient to have angiographically normal coronary arteries with no signs of obstruction or stenosis.

Discussion with the patient to determine the presence of any recent emotional or physical triggers revealed no such events. The patient reported that she was tapered off a selective serotonin reuptake inhibitor (SSRI) medication, specifically escitalopram, two months before the onset of her symptoms due to the complete resolution of her depression and described feeling happier than usual for the past year. She did not endorse any withdrawal symptoms from the tapering of the SSRI. Given the EKG, echocardiogram, and cardiac catheterization findings along with the patient’s troponin levels on admission, she met diagnostic criteria for TTC and was placed on evidence-based medical therapy despite the absence of an identifiable emotional or physical trigger.

## Discussion

While great progress has been made in understanding TTC and its etiology, several questions regarding the pathophysiology of this condition remain unanswered. Many hypotheses have been proposed as potential etiologies. The most widely accepted mechanism involves a surge of plasma catecholamines following an emotionally or physically triggering event leading to a dramatic sympathetic response. Other hypotheses include coronary vasospasm, inflammation, and low estrogen levels [4].

In the patient presented in this case, no strong emotional or physical trigger was identified. The patient reported a generally happy mood and no recent stressful events or traumatic circumstances. It is worth noting that the patient was performing physical activity before the onset of her symptoms. However, her activity level on this day was low to moderate, and typical for her. Generally, physical stressors that have been found to precede a diagnosis of TTC include surgery, severe illness, cocaine use, opiate or alcohol withdrawal, and recovery from general anesthesia [5], none of which this patient had a recent history of. Despite this, the patient did meet diagnostic criteria for TTC according to the revised Mayo Clinic criteria which requires that all of the following be present for diagnosis: presence of myocardial ischemia (ST-segment elevations and/or T-wave inversions), elevation in serum troponin, presence of left ventricular dysfunction, absence of obstructive coronary artery disease diagnosed via angiography, and absence of pheochromocytoma or myocarditis [6]. 

The patient had, however, recently discontinued escitalopram, an SSRI, two months before the onset of her symptoms. Few case reports have described patients who developed TTC following antidepressant withdrawal [7]. While more research must be done to determine a clinically significant connection between antidepressant withdrawal and TTC, cases such as the one reported here may add to the hypothesis that a mechanism unrelated to catecholamine surge may be involved in the development of TTC.

There is evidence that TTC more commonly affects postmenopausal women [4]. The effect of estrogen levels on the development of this condition is still being studied. In studies carried out in rats, estrogen has been found to have cardioprotective effects [8]. While the mechanism by which estrogen provides its cardioprotective effect is complex and not yet fully understood, it is hypothesized that higher levels of estrogen may prevent ischemic reperfusion injury and diminish the effects of catecholamines on the heart, leading to a lower incidence of cardiovascular disease in women compared to men [8,9]. While more research must be conducted in human populations to determine if any significant correlation exists between estrogen levels and cardioprotective effects, these early findings suggest a potential link between postmenopausal status and increased risk for the development of TTC.

The patient presented in this case endorsed a history of asymptomatic left bundle branch block (LBBB) that was found incidentally on a prior preoperative evaluation several years prior. According to the patient, no subsequent workup was done for this diagnosis. Cardiac catheterization during the patient's hospital stay showed no atherosclerosis of coronary arteries. While cardiac magnetic resonance imaging was not done in this patient, this is another helpful tool that provides detailed information about cardiac structure and function and may be used to assess for structural abnormalities to help exclude other differential diagnoses. Although the patient did not endorse previously experiencing symptoms (e.g. palpitations, chest pain, syncope) related to this diagnosis, it is significant to consider this history given her presentation and diagnosis. Research that explores a relationship between pre-existing LBBB and the development of TTC is lacking. While the presence of LBBB in this patient may explain the left ventricular dysfunction noted in the echocardiogram, the significant elevation in troponin levels makes it unlikely that this underlying finding contributed to the patient's acute presentation. Currently, there is currently no clear correlation between LBBB and TTC. 

## Conclusions

TTC remains a fascinating and challenging condition for both clinicians and researchers. Given that approximately a third of patients diagnosed with TTC have no identifiable emotional or physical trigger, there is a clear need for further research regarding the pathogenesis of this condition. Most individuals with TTC who are appropriately diagnosed and treated will fully recover their cardiac function. However, a subset of patients with TTC will continue to have symptoms of persistent heart failure, highlighting the importance of preventing TTC altogether. This report emphasizes the need for further investigation of TTC and the various pathophysiological explanations that may lead to its development. In particular, this report highlights the potential for exploration of the development of TTC in the postmenopausal state and the role that hormonal changes may play in this population. It also underscores the necessity for research into the relationship between SSRI withdrawal and TTC. As our understanding of TTC evolves, there is hope for improved preventative measures, diagnostic methods, and treatment strategies for those affected by this unique cardiac syndrome.
